# Lung lymphatic endothelial cells undergo inflammatory and prothrombotic changes in a model of chronic obstructive pulmonary disease

**DOI:** 10.3389/fcell.2024.1344070

**Published:** 2024-02-19

**Authors:** Anjali Trivedi, Tyler M. Lu, Barbara Summers, Kihwan Kim, Alexander J. Rhee, Sean Houghton, Derek E. Byers, Raphaël Lis, Hasina Outtz Reed

**Affiliations:** ^1^ Department of Medicine, Division of Pulmonary and Critical Care, Weill Cornell Medicine, New York, NY, United States; ^2^ Ansary Stem Cell Institute, Division of Regenerative Medicine, Department of Medicine, Weill Cornell Medicine, New York, NY, United States; ^3^ Ronald O. Perelman and Claudia Cohen Center for Reproductive Medicine, Weill Cornell Medicine, New York, NY, United States; ^4^ Molecular and Cellular Biology Program, SUNY Downstate School of Graduate Studies, Brooklyn, NY, United States; ^5^ Department of Medicine, Division of Pulmonary and Critical Care, Washington University School of Medicine, St. Louis, MO, United States; ^6^ Department of Cell and Developmental Biology, Weill Cornell Medicine, New York, NY, United States

**Keywords:** chronic obstructive pulmonary disease, cigarette smoke, lymphatic endothelial cells, lymphatic vasculature, inflammation

## Abstract

The lymphatic vasculature regulates lung homeostasis through drainage of fluid and trafficking of immune cells and plays a key role in the response to lung injury in several disease states. We have previously shown that lymphatic dysfunction occurs early in the pathogenesis of chronic obstructive pulmonary disease (COPD) caused by cigarette smoke (CS) and that this is associated with increased thrombin and fibrin clots in lung lymph. However, the direct effects of CS and thrombin on lymphatic endothelial cells (LECs) in COPD are not entirely clear. Studies of the blood vasculature have shown that COPD is associated with increased thrombin after CS exposure that causes endothelial dysfunction characterized by changes in the expression of coagulation factors and leukocyte adhesion proteins. Here, we determined whether similar changes occur in LECs. We used an *in vitro* cell culture system and treated human lung microvascular lymphatic endothelial cells with cigarette smoke extract (CSE) and/or thrombin. We found that CSE treatment led to decreased fibrinolytic activity in LECs, which was associated with increased expression of plasminogen activator inhibitor 1 (PAI-1). LECs treated with both CSE and thrombin together had a decreased expression of tissue factor pathway inhibitor (TFPI) and increased expression of adhesion molecules. RNA sequencing of lung LECs isolated from mice exposed to CS also showed upregulation of prothrombotic and inflammatory pathways at both acute and chronic exposure time points. Analysis of publicly available single-cell RNA sequencing of LECs as well as immunohistochemical staining of lung tissue from COPD patients supported these data and showed increased expression of inflammatory markers in LECs from COPD patients compared to those from controls. These studies suggest that in parallel with blood vessels, the lymphatic endothelium undergoes inflammatory changes associated with CS exposure and increased thrombin in COPD. Further research is needed to unravel the mechanisms by which these changes affect lymphatic function and drive tissue injury in COPD.

## Introduction

Chronic obstructive pulmonary disease (COPD) is a devastating and phenotypically heterogeneous disease for which there are currently no therapies that halt disease progression. COPD is most commonly caused by cigarette smoke (CS) exposure, but we do not fully understand the pathogenic mechanisms that drive the disease in these patients. The blood vasculature has been a well-studied subject of investigation in COPD (Lu et al., 2018; Polverino et al., 2018). COPD patients have a prothrombotic phenotype in the blood vasculature that is characterized by increased thrombin that overwhelms the inhibitory activity of the tissue factor pathway inhibitor (TFPI) (Cella et al., 2001; Polosa et al., 2008; Undas et al., 2011; Kyriakopoulos et al., 2021). In addition, blood vascular dysfunction in COPD is caused by a detrimental effect of CS on endothelial cells due to oxidative stress and chronic inflammation (Peinado et al., 1998; Polverino et al., 2018). In this way, the effects of increased thrombin generation and CS exposure on blood endothelial cells may both play distinct roles in vascular dysfunction in COPD. That endothelial cell dysfunction occurs early in the disease process (Peinado et al., 1998) and is associated with disease severity (Eickhoff et al., 2008; Moro et al., 2008; Minet et al., 2012) has supported a “vascular hypothesis” in the pathogenesis of COPD, where changes in the blood vasculature are thought to play an important role in disease progression (Liebow, 1959; Kasahara et al., 2000; Cella et al., 2001; Polosa et al., 2008; Undas et al., 2011; Voelkel et al., 2011; Lu et al., 2018; Polverino et al., 2018; Hisata et al., 2021; Kyriakopoulos et al., 2021).

Distinct from the blood vasculature, the lymphatic vasculature also forms a network in the lungs and plays an important, though often overlooked, role in lung function. The lymphatic vasculature maintains lung homoestasis by regulating fluid clearance, trafficking of immune cells, and coordinating the inflammatory response (Stump et al., 2017; Trivedi and Reed, 2023). Lymphatic endothelial cells (LECs) line lymphatic vessels and are involved in crosstalk with trafficking leukocytes as well as responses to lung injury. Though current evidence suggests that lymphatic dysfunction plays a role in the pathogenesis of many lung diseases, it is not known whether LEC injury occurs in COPD (Ebina, 2008; El-Chemaly et al., 2008; Stump et al., 2017). We and others have previously reported abnormal lung lymphatic function in the setting of COPD (Hardavella et al., 2012; Mori et al., 2013a; Summers et al., 2022). We found that CS exposure results in fibrin-rich clots in the lung lymphatics in both mouse models and human COPD (Summers et al., 2022). Furthermore, we also found increased thrombin in lung-draining lymph after CS exposure in these mice, suggesting that the lymphatics are exposed to a prothrombotic milieu in this setting. Thus, similarly to the blood endothelium, where CS exposure and increased thrombin affect the function of these vessels in COPD, the lung lymphatics may also be subject to thrombin and CS-mediated changes, though this has not been previously explored.

In this study, we used both *in vitro* and *in vivo* models as well as human lung tissue to investigate the changes in the lymphatic endothelium that occur in COPD. We found that CS exposure decreased the fibrinolytic activity of LECs, which was associated with increased plaminogen activator inhibitor 1 (PAI-1). Thrombin exposure to LECs *in vitro* led to increased expression of inflammatory markers such as intracellular adhesion molecule 1 (ICAM-1) and vascular adhesion molecule 1 (VCAM-1) while decreasing the expression of TFPI. In an *in vivo* mouse model of COPD, RNA sequencing of LECs isolated from mice exposed to CS also showed increased pathways of inflammation and increased expression of leukocyte adhesion molecules. Immunohistochemistry of human tissue showed similar results, with increased expression of inflammatory markers in lymphatics in COPD patients, compared to controls. These results suggest that in parallel with the blood vasculature, CS- and thrombin-mediated changes in the lymphatic endothelium may be a previously unrecognized aspect of COPD pathogenesis.

## Results

### CSE decreases LEC fibrinolytic activity *in vitro*

**FIGURE 1:**
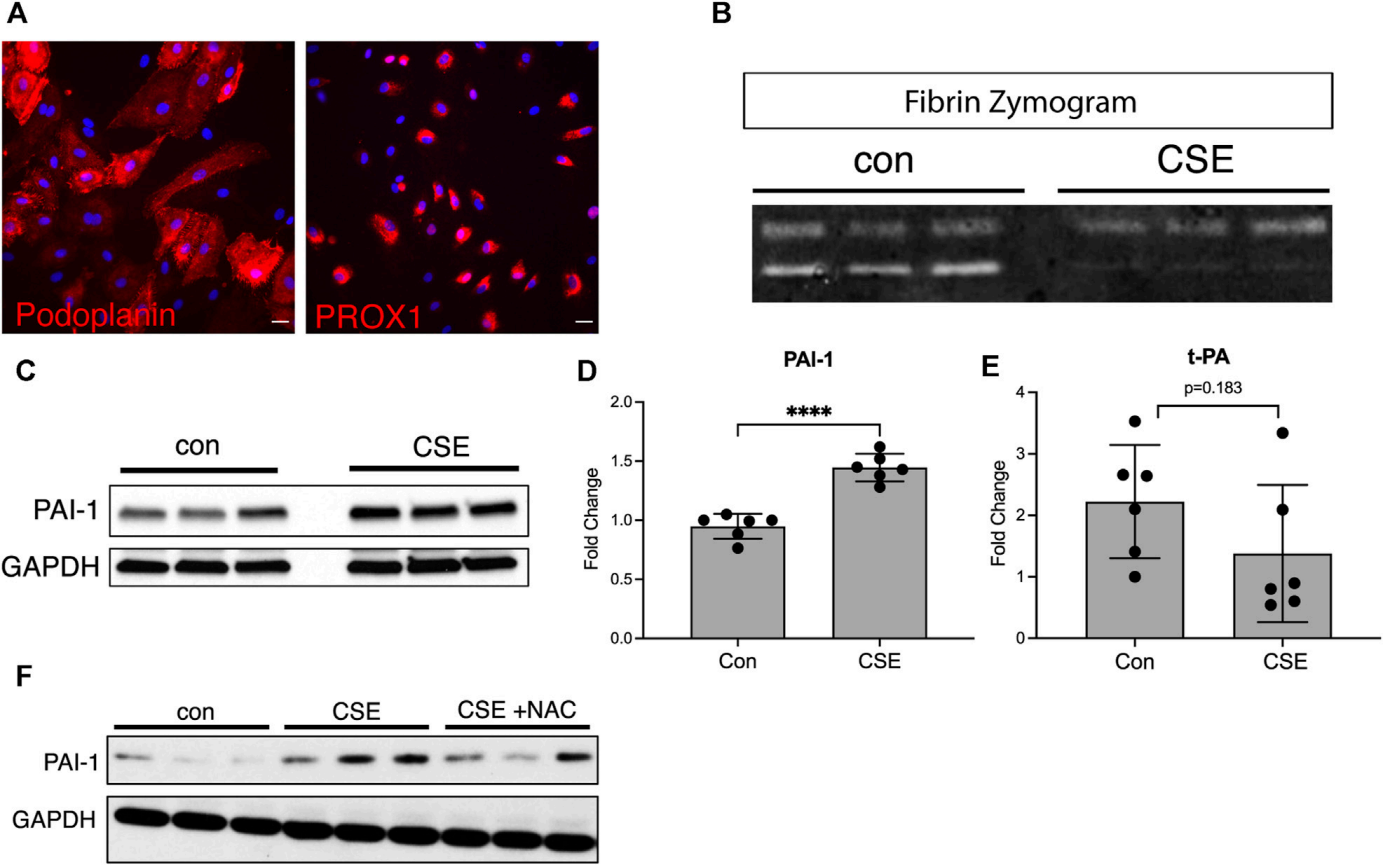
CSE decreases the fibrinolytic activity of LECs *in vitro.* **(A)** Immunocytochemical staining of cultured human microvascular lymphatic endothelial cells (LECs) for the human lymphatic markers, PROX1 and podoplanin (red). **(B)** Fibrin gel zymogram using supernatants from control or CSE-treated LECs. **(C)** Western blot for PAI-1 using cell lysates from control or CSE-treated LECs. GAPDH was used as a loading control on the same blot. **(D,E)** Expression of PAI-1 and t-PA by quantitative PCR using lysates from control or CSE-treated LECs and normalized to GAPDH. **(F)** Western blot using lysates from cells treated with CSE with and without co-incubation with *N*-acetylcysteine (NAC). GAPDH was used as a loading control on the same blot. Values are expressed as means ± SD. *p*-value calculated by Student’s t-test, **** *p* < 0.0001, ns = not significant. Data representative of two independent experiments performed in triplicate.

In normal settings, the lymphatic vasculature is inherently antithrombotic due to the high level of fibrinolytic activity in the LECs and the relatively low levels of prothrombotic factors in lymph (Lippi et al., 2012). Lymphatic thrombosis is, therefore, a rare event and implies that there has been a shift in the balance of prothrombotic factors and fibrinolytic activity. We determined whether cigarette smoke extract (CSE) alters the fibrinolytic activity of LECs using an *in vitro* culture system with human lung microvascular endothelial cells to determine the effects of cigarette smoke extract on LECs. Staining for the human lymphatic markers PROX1 and podoplanin verified that these cells maintained their lymphatic identity in culture (Figure 1A). We performed fibrin zymography as previously described (Kim et al., 1998; Choi and Kim, 2000) using supernatants from LECs treated with the CSE compared to those from controls. We found that supernatants from control LECs demonstrated robust fibrinolytic activity, as expected, as demonstrated by the bands on the zymogram gel formed by protease activity (Figure 1B). However, supernatants from CSE-treated LECs showed decreased protease activity in the zymogram (Figure 1B). The fibrinolytic state of LECs is driven, in part, by relatively low levels of plasminogen activator inhibitor-1 (PAI-1), which inhibits the activity of plasminogen activators tPA and uPA to create a prothrombotic state (Lippi et al., 2012; Sillen and Declerck, 2020). We found increased expression of PAI-1 in LECs treated with CSE compared to those of the controls by both Western blot and quantitative PCR (Figures 1C, D). CSE treatment also led to a small but not statistically significant decrease in the expression of the fibrinolytic protease tPA (Figure 1E), but it did not alter the expression of other endothelial homeostatic factors such as endothelial protein C receptor (EPCR) or thrombomodulin (THBD) (Supplemental Figure S1). We then determined whether the increase in expression of PAI-1 in LECs after CSE exposure is due to oxidative stress. We found that treatment of LECs with the antioxidant *N*-acetylcysteine partially inhibited the upregulation of PAI-1 after CSE exposure (Figure 1F), suggesting that oxidative stress is indeed a cause of decreased fibrinolytic activity in LECs.

### Thrombin leads to an inflammatory phenotype of LECs *in vitro*

**FIGURE 2:**
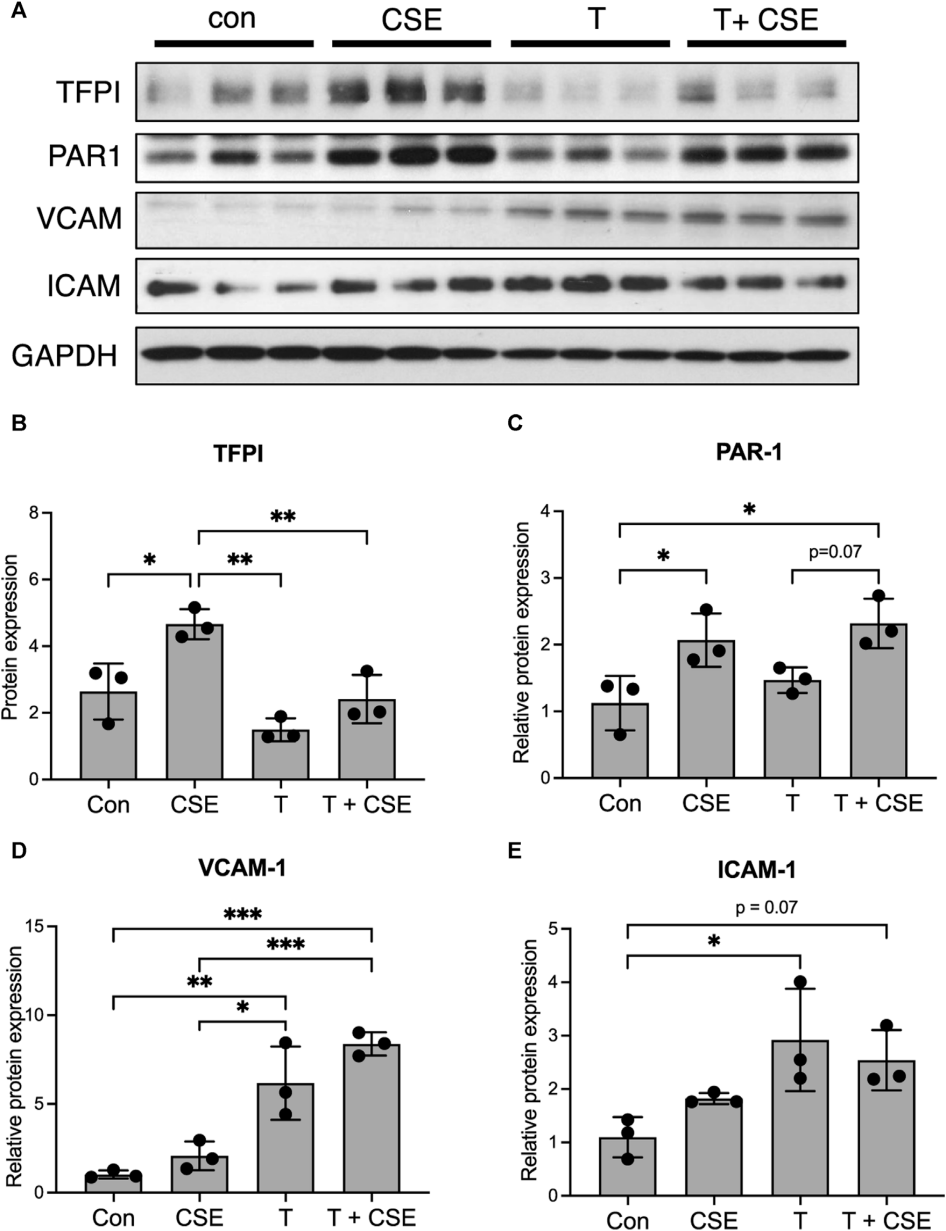
Thrombin leads to an inflammatory phenotype in LECs *in vitro.* **(A)** Western blot for TFPI, PAR-1, VCAM, and ICAM in lysates from LECs grown in culture and treated with CSE overnight and/or thrombin for 10 min. GAPDH was probed on the same membrane as a loading control. **(B–E)** Relative densitometric graphs of Western blots for TFPI, PAR-1, VCAM, and ICAM. All values are expressed as means ± SD. *p*-value calculated by ANOVA. *p < 0.05, **p < 0.01, and ***p < 0.001. Scale bars = 25 μm. Data are representative of two independent experiments performed in triplicate.

Our previous work has shown that CS exposure results in increased thrombin in lymph draining in the lung lymphatics, which may be due to the overall increase in systemic thrombin that is seen with CS exposure in COPD (Jankowski et al., 2011; Undas et al., 2011). Given the effects of thrombin on endothelial cell function (Rabiet et al., 1994; Rabiet et al., 1996; O'Brien et al., 2000; Minami et al., 2004), we hypothesized that the lung lymphatics may be affected not only by CS itself but also by the increased presence of thrombin that LECs are exposed to after CS exposure. We treated LECs with CSE and thrombin and assessed the expression of thrombotic markers and leukocyte adhesion molecules. We found that CSE alone led to an increased expression of the tissue factor pathway inhibitor (TFPI) and the thrombin receptor protease activated receptor-1 (PAR-1) (Figures 2A–C). While CSE increased the expression of TFPI, LECs exposed to thrombin at decreased expression of TFPI (Figures 2A, B). In addition, LECs treated with thrombin had an increased expression of VCAM-1 and ICAM-1 compared to those treated with CSE alone (Figures 2A, D, and E).

### RNA sequencing reveals upregulated leukocyte adhesion molecules and inflammatory markers in lung LECs from CS-exposed mice

**FIGURE 3:**
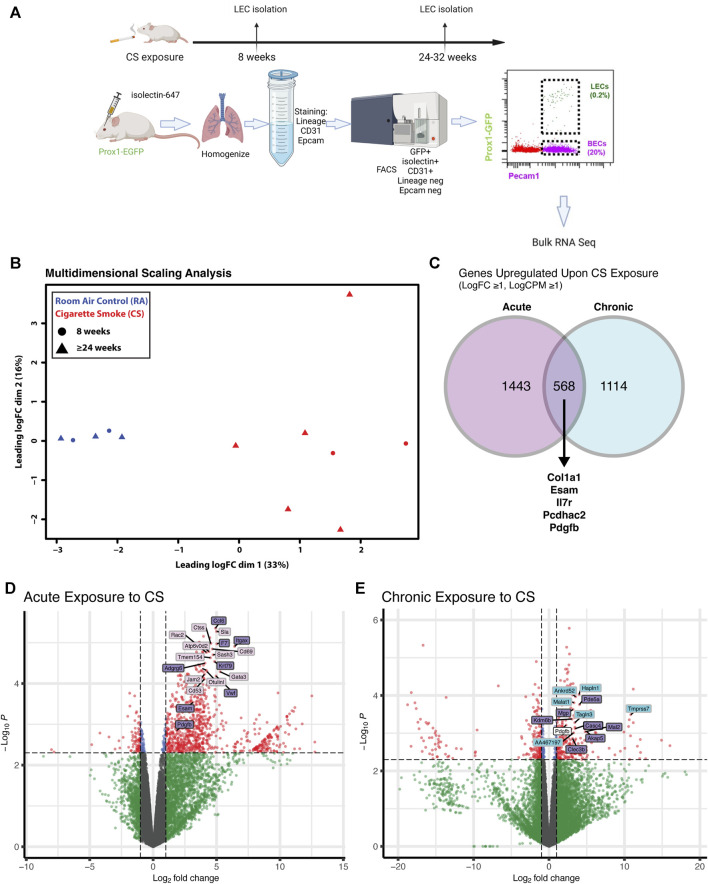
Transcriptional profiling of isolated lung LECs from mice after acute and chronic CS exposure. **(A)** Experimental design isolation of lung LECs from *PROX1-EGFP* lymphatic reporter mice at time points after CS exposure starting at 6 weeks of age. **(B)** Multidimensional scaling analysis of FACS isolated LECs after acute (8 weeks) and chronic (≥24 weeks) exposure to CS *in vivo,* highlighting the significant effect of smoke on global transcriptomic profiles. **(C)** Venn diagram of genes significantly upregulated (LogFC ≥1, LogCPM ≥1) in LECs after acute and chronic exposure to CS showing examples of commonly upregulated genes. **(D)** Volcano plot highlighting significantly differentially expressed genes in LECs after acute exposure to CS, highlighting unique and commonly upregulated genes. **(E)** Volcano plot highlighting significantly differentially expressed genes in LECs after chronic exposure to CS, highlighting unique and commonly upregulated genes.

We next determined whether the changes we have observed *in vitro* are also present in LECs in mice after CS exposure. To determine the short- and long-term effects of CS on the lymphatic endothelium *in vivo*, we used a novel technique of lung LEC isolation for downstream analysis. Lymphatic reporter mice, in which all LECs are labeled by GFP (*Prox1-EGFP*) (Choi et al., 2011), were exposed to CS using a whole-body system for acute (8 weeks) or chronic (24 weeks or more) time points, and their LECs were compared to lung LECs from age-matched control mice. Immediately prior to sacrifice, the mice were injected with fluorescently labeled isolectin for intravital labeling of all endothelial cells, and single-cell suspensions from lung homogenates were stained for FACS with negative selection for the epithelial marker EpCAM and a hematopoietic lineage marker cocktail, as well as positive selection for GFP and CD31 (Figure 3A). This technique led to a pure population of LECs that was roughly 0.2% of total lung cells, which is in line with previous single-cell data sets (Schupp et al., 2021).

**FIGURE 4:**
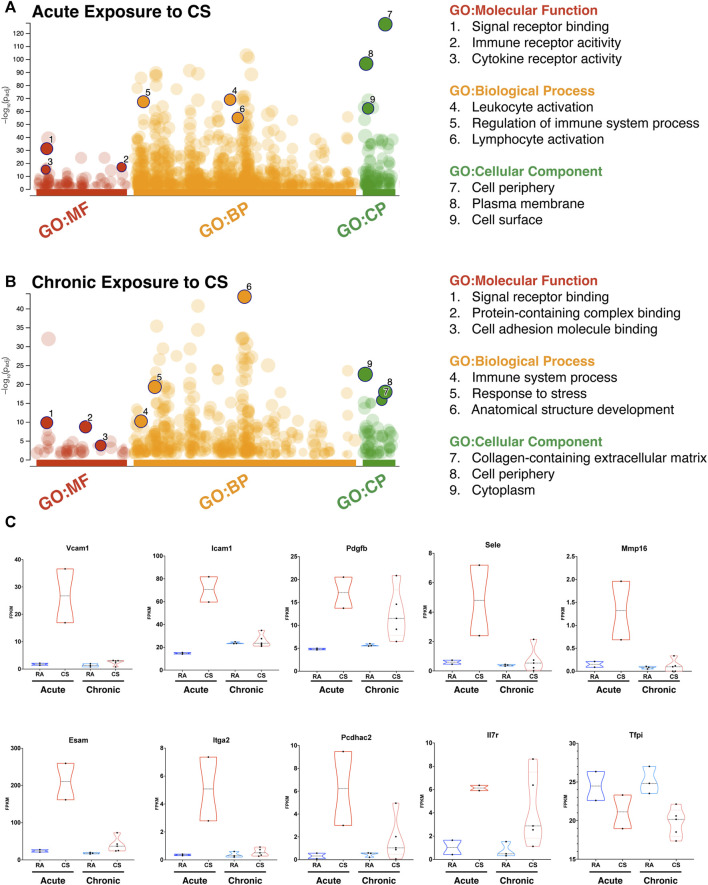
RNA sequencing of lung LECs of CS-exposed mice reveals prothrombotic and inflammatory transcriptional changes. **(A)** Gene set enrichment analysis using uniquely upregulated genes in LECs after acute exposure to CS, highlighting corresponding molecular pathways and components. **(B)** Gene set enrichment analysis using uniquely upregulated genes in LECs after chronic exposure to CS, highlighting corresponding molecular pathways and components. **(C)** Violin plots demonstrating the expression (FPKM) of select genes from leukocyte adhesion and inflammatory pathways between LECs after acute or chronic exposure to CS and their age-matched controls (RA).

**FIGURE 5:**
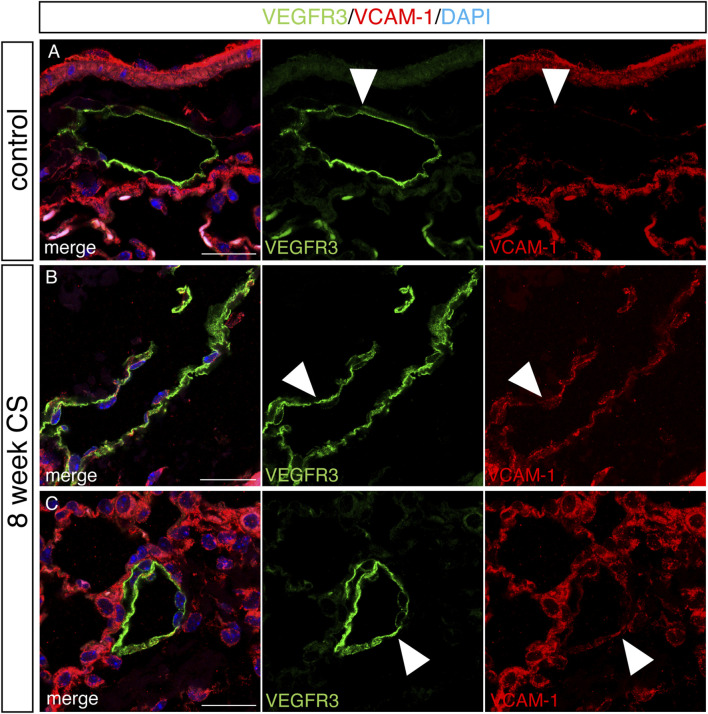
Increased staining for VCAM in lung lymphatics in a mouse model of COPD. Mice were exposed to full body CS for 8 weeks, and the lungs were harvested for immunohistochemical analysis. **(A–C)** Representative images of immunohistochemical staining of lung tissue for mouse lymphatic marker VEGFR3 (green), VCAM (red), and counterstained with DAPI in control mice **(A)** and mice exposed to CS for 8 weeks **(B,C)**. Lymphatics (green) and VCAM (red) are indicated by arrow heads. Scale bars = 25 μm. Representative images of lung tissue from n = 5 control and n = 5 CS-exposed mice.

We analyzed LECs after acute and chronic CS exposure using RNA sequencing and compared it to lung LECs from age-matched controls. We found significant alterations in the transcriptome of LECs after both acute and chronic CS exposure (Figures 3B–E). Interestingly, we did not observe many transcriptional changes in control room air LECs isolated at 8 weeks compared to those isolated at greater than 24 weeks (Figure 3B), suggesting that CS confers more changes in LECs than age alone. In addition, while there were many transcriptional changes that were unique to acute and chronic CS exposure, there were also many genes that were altered at both time points (Figures 3C–E). Notably, pathways associated with cell adhesion and leukocyte migration as well as those that are predicted to play a role in inflammation and immune cell recruitment were upregulated in CS-exposed LECs at both acute and chronic exposure time points (Figures 4A, B). In addition, we observed the upregulation of pathways associated with interactions with the extracellular matrix, particularly after chronic CS exposure (Figures 4A, B). Key genes crucial for leukocyte migration and adhesion, including *Vcam1*, *Icam1*, endothelial cell-specific adhesion molecule (*Esam*), and endothelial selectin (*Sele*), were also significantly upregulated in LECs from CS-exposed mice compared to control LECs (Figure 4C). Additionally, we observed CS-induced upregulation of chemokines and markers of inflammation, including platelet-derived growth factor B (*Pdgfb*), matrix metalloproteinase 16 (*Mmp16*), and interleukin receptor-17 (*IL-17r*), in CS-exposed LECs compared to control LECs (Figure 4C). Interestingly, we observed a decreased expression of *Tfpi* in LECs from CS-exposed mice, similar to what was seen in LECs exposed to thrombin *in vitro* (Figure 4C). Immunohistochemistry of lung tissue from mice exposed to CS for 8 weeks showed increased staining of VCAM-1 on the lymphatic vessels compared to room air control mice (Figures 5A–C). Thus, *in vivo* transcriptomic analysis supports our *in vitro* data and suggests that LECs in CS-exposed mice undergo transcriptional changes that may result from the effects of CS-induced thrombin in the lung lymphatic vasculature. We studied publicly available single-cell RNA sequencing data of lung tissue obtained from the explanted lungs of patients with advanced COPD requiring lung transplant and control donor lungs (Sauler et al., 2022). Interestingly, we also found a relatively high level of expression of *Tfpi* in LECs, which was then decreased in LECs from COPD patients, identical to what we observed in LECs from CS-exposed mice (Supplementary Figure S2). The expression of many of the genes that we observed using RNA sequencing of isolated LECs from mice was not detected in LECs from this dataset of whole lung tissue, likely due to scarcity of LECs in the lungs making detection of these transcripts difficult without specific isolation of these cells. However, we did find an increased expression of *Mmp16* and *Pdgfb* in LECs from COPD patients compared to those from controls, though the expression of these genes was low.

### Increased staining for VCAM-1 and SMA associated with lung lymphatics from patients with end-stage COPD

**FIGURE 6:**
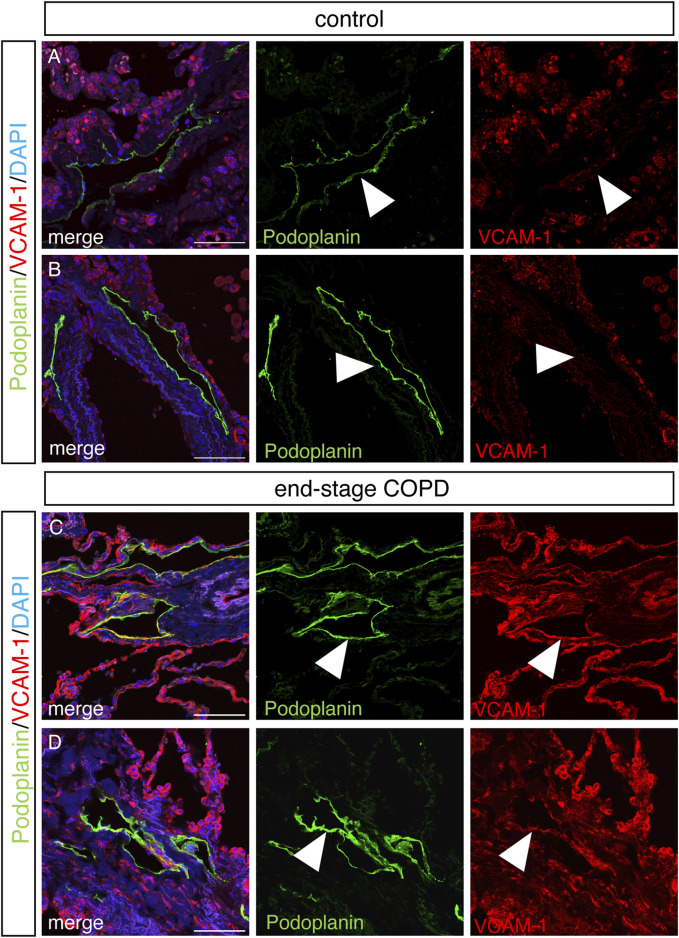
Increased staining for VCAM in lung lymphatics in end-stage COPD. Representative images of immunohistochemical staining of human lung tissue stained for VCAM (red) and podoplanin (green) from control **(A,B)** and end-stage COPD **(C,D)** patients and counterstained with DAPI. Lymphatics (green) and VCAM (red) are indicated by arrow heads. Scale bars = 25 μm. Representative images of n = 5 control and n = 10 end-stage COPD patients.

**FIGURE 7:**
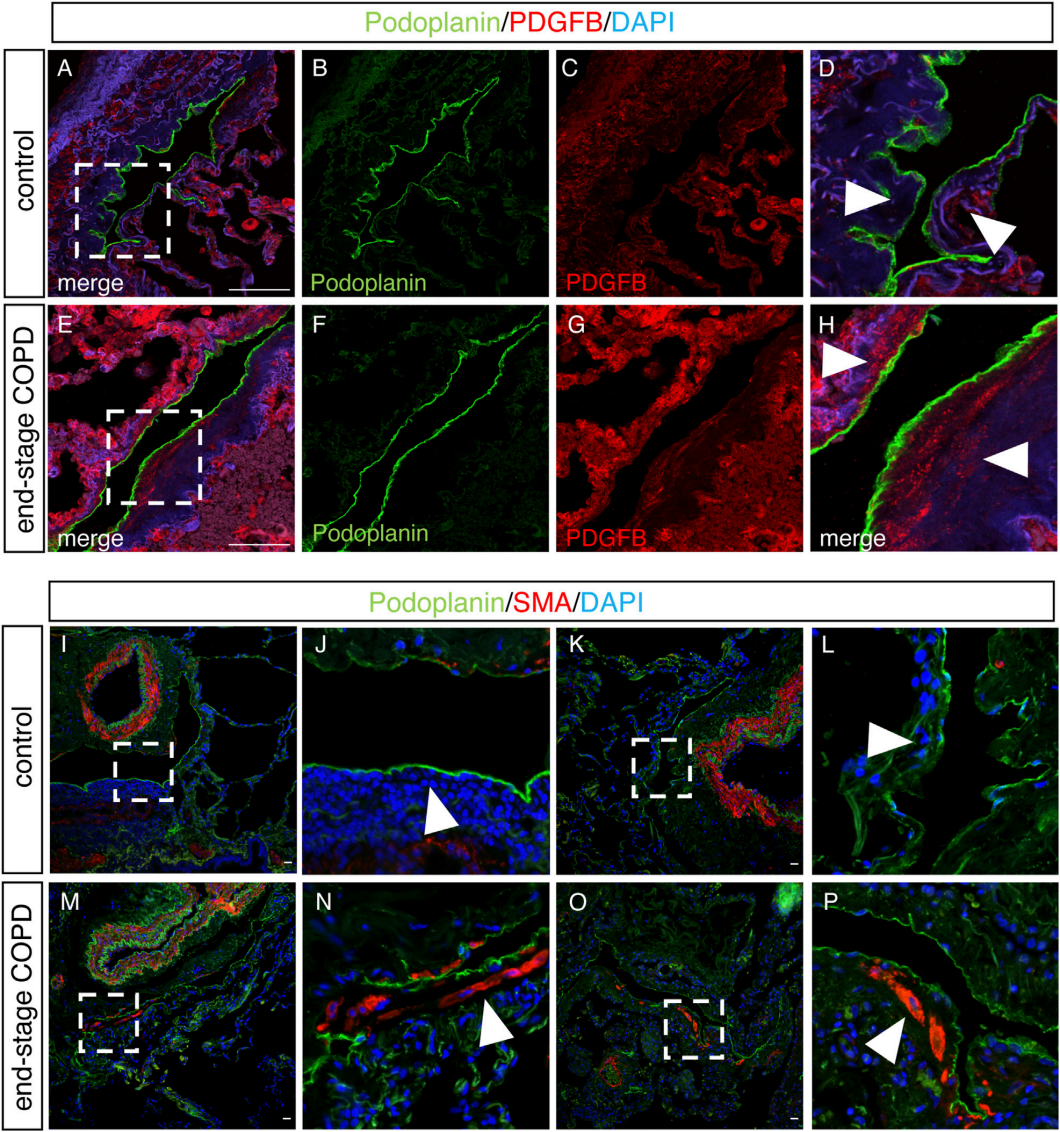
Upregulation of PDGFB and SMA on lung lymphatics in end-stage COPD. **(A–E)** Representative images of immunohistochemical staining of lung tissue from control or end-stage COPD for the human lymphatic markers podoplanin (green) and PDGFB (red). **(D,H)** Magnified image of the area indicated in images A and E, respectively. Arrowheads demonstrating the areas of the extracellular matrix around lymphatic vessels. **(I–P)** Representative immunohistochemical images of human lung tissue from control or end-stage COPD patients for the lymphatic markers podoplanin (green) and SMA (red). **(J,L)** Magnified image of the area indicated in I and K, with arrowheads indicating areas around lymphatic vessels. **(N,P)** Magnified image of the areas indicated in M and O, with arrowheads indicating the areas around lymphatic vessels. Scale bars = 25 μm. Representative images of n = 5 control and n = 10 end-stage COPD patients.

We next sought to determine whether some of the changes we observed in lung LECs from mice were also present in the lymphatics from patients with COPD using immunohistochemical analysis on lung tissue samples from patients with end-stage COPD compared to lung tissue from control nonsmokers without lung disease. Staining for the human lymphatic marker podoplanin and VCAM-1 revealed a robust expression of VCAM-1 in the lymphatics in end-stage COPD patients that was not present in control lymphatics (Figures 6A–D). Interestingly, we also observed that VCAM-1 was more highly expressed throughout the lung tissue from COPD patients compared to controls, likely reflecting the known upregulation of VCAM-1 in the blood endothelium in COPD (Borek et al., 2023). We also investigated whether the expression of PDGFB was increased in lung lymphatics in COPD patients compared to controls, as this was seen in CS-exposed mice at acute and chronic exposure time points by RNA sequencing. Using immunohistochemistry, we found evidence of increased staining for PDGFB around the lymphatic vessels in the lungs of COPD patients (Figures 7E–H) compared to control patients (Figures 7A–D). This PDGFB staining was associated with the basement membrane and extracellular matrix of the lymphatic vessels, similar to previous reports (Wang et al., 2017) where PDGFB retention is necessary for its role in accessory cell recruitment to these vessels. Normal lung lymphatics have minimal smooth muscle cell (SMC) coverage, as opposed to lymphatics in other organs, and ectopic recruitment of SMC lymphatics is a marker of dysfunction in these vessels (Meinecke et al., 2012; Sweet et al., 2015; Reed et al., 2019). Interestingly, we found increased staining for smooth muscle actin-positive SMCs associated to the lung lymphatics in lung tissue from patients with COPD compared to controls (Figures 7I–P). Single-cell RNA sequencing data suggested that PDGFB is increased in the lungs in COPD, though LECs are likely not the major source (Supplementary Figure S2). These data suggest that increased PDGFB driven by LECs and other cell types may drive SMC recruitment to these vessels in COPD, though this has not been definitely shown.

## Discussion

In this study, we build on our previous studies on mice and human tissue that found increased thrombin, lymphatic thrombosis, and impaired lymphatic flow in COPD and now explore the molecular signature of LECs exposed to CS and thrombin *in vitro* and *in vivo*. Using a cell culture system, we found that CS exposure led to decreased fibrinolytic activity in LECs, which was associated with an increased expression of the antifibrinolytic PAI-1. LECs exposed to thrombin had an increased expression of PAR-1, VCAM-1, and ICAM-1, as well as a decreased expression of TFPI. These *in vitro* findings were supported by RNA sequencing of LECs isolated from a mouse model of COPD, where we found decreased *Tfpi* and upregulation of the pathways of inflammation in these cells. Interestingly, analysis of single-cell RNA sequencing data from human end-stage COPD (Sauler et al., 2022) also demonstrated that LECs are a prominent source of TFPI in the lungs and that the expression of TFPI is decreased in LECs in COPD. As TFPI is the major inhibitor of thrombosis in both the blood and lymphatic endothelium (Undas et al., 2011; Lippi et al., 2012), decreased expression of TFPI in the setting of increased expression of PAR-1, VCAM-1, and ICAM-1 suggests a prothrombotic and inflammatory phenotype of LECs, driven by increased thrombin with CS exposure.

Thrombin is increased both systemically and in lung lymphatics after CS exposure and in COPD patients (Undas et al., 2011; van der Vorm et al., 2020; Summers et al., 2022). Our results suggest that increased thrombin after CS exposure may mediate changes in LECs that promote thrombosis and decrease lymphatic function. Many of the factors that we observed to be upregulated in LECs after CS exposure, including VCAM-1, ICAM-1, and PDGFB, have been shown to be downstream of thrombin signaling through the receptor PAR-1 in the blood endothelium (Minami et al., 2004). PAR-1 is the most prominent receptor for thrombin in endothelial cells in both mice and humans (O'Brien et al., 2000; Minami et al., 2004; Kataoka et al., 2003), and signaling through PAR-1 is a well-studied cause of blood endothelial dysfunction (Colotta et al., 1994; Rabiet et al., 1994; Rabiet et al., 1996; Kaplanski et al., 1998; Marin et al., 2001; Miho et al., 2005; Okada et al., 2006). PAR-1 polymorphisms have been implicated in the development of COPD (Yun and Sang, 2015) as well as protection from exacerbations (Plate et al., 2014). However, PAR-1 likely functions in a very cell type-specific and context-dependent manner, (Howell et al., 2005; Kaneider et al., 2007; Schouten et al., 2012; Aerts et al., 2013; Kager et al., 2014; Mercer et al., 2014). Future studies on whether and how thrombin signaling through PAR-1, specifically in LECs, affects lymphatic function and lung injury after CS exposure are warranted.

Immunohistochemistry of human tissue supported the findings of our *in vitro* and mouse model studies and revealed increased VCAM-1 and PDGF associated with lymphatics in COPD patients compared to control patients. Increases in these markers correlate with vascular dysfunction and a prothrombotic state in the blood endothelium in COPD (Cella et al., 2001; Moro et al., 2008). Our results suggest that a similar pattern of injury occurs in the lymphatic endothelium, where we have seen decreased drainage and thrombosis after CS exposure (Summers et al., 2022). Furthermore, PDGFB is associated with remodeling and lymphatic dysfunction (Petrova et al., 2004; Tammela et al., 2007; Meinecke et al., 2012; Wang et al., 2017; Folestad et al., 2018), and aberrant recruitment of SMCs to the lung lymphatics is a marker of dysfunction, as there are typically minimal SMA^+^ cells around lung lymphatics in contrast to lymphatics of other organs (Meinecke et al., 2012; Kretschmer et al., 2013; Reed et al., 2019). While these immunohistochemical data are notably limited by not being quantitative, they agree to some extent with transcriptional data from single-cell sequencing, and the expression patterns we observed are supported by our *in vitro* data and our analysis of LECs in mice. Importantly, though they play a pivotal role in lung homeostasis, LECs are a relatively rare cell population (∼0.2% of total lung cells in both the mouse and humans). Therefore, even single-cell RNA sequencing of whole lungs may fail to capture important transcriptional changes in these cells, compared to an analysis of an isolated LECs, as we have done here in CS-exposed mice. Given that we and others have clarified the lung-specific markers of LECs (Baluk and McDonald, 2008; Reed et al., 2019), sorting of these cells to generate an enriched population for downstream analysis is warranted and likely to uncover novel insights into how changes in these cells may play a role in disease pathogenesis.

The expression of adhesion molecules such as VCAM-1 and ICAM-1 on the lymphatic endothelium is important for leukocyte migration in collecting lymphatic vessels in the settings of inflammation (Johnson et al., 2006; Arasa et al., 2021). We have previously shown decreased lymphatic drainage and leukocyte trafficking after CS exposure; therefore, further studies should elucidate whether there are lung-specific roles for VCAM-1 and other adhesion molecules in lymphatic function after CS exposure. Importantly, we observed that CS exposure induced significant inflammatory changes in the transcriptome of LECs at both acute and chronic exposure time points, but these changes were most profound at the 8-week time point. This temporal regulation of gene expression likely reflects the acute and adaptive responses of the lymphatic endothelium to inflammation, as opposed to the secondary changes as a result of lung parenchymal injury, which typically does not occur until after 6 months of CS exposure in this model (Vlahos and Bozinovski, 2014; De Cunto et al., 2018; Summers et al., 2022). Lymphatic dysfunction causes increased inflammation that promotes lung injury and inflammation in COPD (Mori et al., 2013b; Reed et al., 2019; Summers et al., 2022). The results shown here suggest that direct effects of CS and thrombin on the lymphatic endothelium may promote decreased lymphatic drainage, decreased leukocyte trafficking, and thrombosis that are seen after CS exposure in mouse models and human tissue. The lymphatic vasculature may be susceptible to inflammatory changes in the lungs after CS exposure that occur prior to tissue remodeling and, therefore, represent an attractive target for novel therapeutic interventions that could affect the disease trajectory.

Taken together, our data suggest that lymphatic dysfunction in COPD is characterized by the effects of CS and increased thrombin on the lymphatic endothelium, though the mechanism for this remains to be determined. Future studies should be undertaken to understand how lymphatic dysfunction in COPD may drive tissue injury and disease pathogenesis.

## Methods

### Mouse cigarette smoke exposure model

*Prox1-EGFP* mice have been previously described (Choi et al., 2011). C57Bl/6 or *Prox1-EGFP* mice were exposed to cigarette smoke for 8 weeks (acute) or ≥24 weeks [using a whole-body exposure system (TE-10) (supplied by Teague Enterprises)] with 3R4F composition cigarettes (University of Kentucky Center for Tobacco Reference Products). Mice were exposed to CS (∼150 mg/m^3^) 5 days a week for abovementioned time points. Control mice exposed to room air alone were age-matched and identically housed in the same room and on the same rack. Mice were housed in the Weill Cornell animal facility in 12/12 h light/dark cycles with *ad libitum* access to water and food. At the respective time points, CS-exposed C57Bl/6 mice or *Prox1-EGFP* mice and age-matched room air control mice were identically housed in the animal facility. We used both male and female mice in our experimental and control groups.

### *In vitro* cell culture system

Human lung microvascular lymphatic endothelial cells (Lonza) were cultured in EGM-2 media with a EGM-2 Endothelial Cell Growth Medium-2 BulletKit (Lonza, CC-3162) at 37°C and 5% CO_2_ to ensure confluence and then plated at a density of 187,000 cells per well. The cells were then starved in EGM-2 without added supplemental growth factors or serum. For cells treated with cigarette smoke extract (CSE) alone, 2% CSE in DMEM was then added to EGM-2 media without supplemental growth factors overnight. For cells treated with CSE and thrombin, 2% CSE was added to cells initially for 6 h. After 6 h, 1U/μL thrombin (Sigma, T4393) was added to the wells for 10 min. All the wells were then washed with serum-free media, and media with 2% CSE was again added to cells and incubated overnight. Cells treated with thrombin alone were treated for 10 min, washed two times in serum-free media, and incubated overnight. After overnight incubation for all wells, cell lysates and supernatants were collected using RIPA and protease inhibitors the next morning for protein and RNA isolation. For co-incubation with N-acetylcysteine (NAC), LECs were cultured and plated as discussed above. All the wells were washed with EGM-2 media without added supplemental growth factors or serum. The control cells were left in serum-free media overnight. For cells treated with CSE alone, 2% CSE in serum-free media was then added and incubated overnight. For cells treated with CSE and NAC, a freshly prepared 5 µM NAC solution (Sigma, A9165) in serum-free media was added to the wells for 2 h. CSE (2%) in serum-free media was then added to these wells and incubated overnight. After overnight incubation for all wells, cell lysates and supernatants were collected using RIPA and protease inhibitors the next morning for protein and RNA isolation.

### Fibrin zymography

Zymography was performed using modifications of previously described protocols (Kim et al., 1998; Choi and Kim, 2000). The supernatants obtained from both untreated (control) and 2% CSE-treated LECs were run on a 12% SDS-PAGE gel containing thrombin and fibrinogen, with tissue plasminogen activator (tPA) run as a positive control for fibrinolysis within the gel. The gel was washed in Triton-X and incubated overnight at 37° in a CaCl_2_-containing Tris-HCL buffer. Staining was performed with Coomassie Blue, and the gel was de-stained in methanol and acetic acid to reveal areas of fibrin cleavage.

### Western blotting

Western blotting was performed according to the standard protocols and probed with PAI-1 (R&D Systems, MAB1786), TFPI (Abcam, ab180619), PAR-1 (Sigma, sab4500823), VCAM1 (Novus, NBP2-29413), ICAM1 (Novus, NBP2-67518), and GAPDH (Santa Cruz Biotechnology, SC365062). Blots were imaged using a Konica Minolta SRX-101A Medical film processor and quantified with ImageJ software. The scanned Western blot images were imported into the ImageJ software. Regions of interest (ROIs) were defined around the bands corresponding to the protein of interest and the reference protein, GAPDH, by selecting the “rectangle tool.” Densitometric analysis was then conducted with the ImageJ using the “measure” option to quantify the intensities of these bands. The obtained target protein intensities were then normalized to the corresponding GAPDH bands, which served as an internal control.

### Quantitative PCR

Cell lysates from HMVLECs were harvested and homogenized for RNA isolation. Total RNA from cell lysates was isolated using an RNEasy Kit (Qiagen). cDNA was made according to the manufacturer’s instructions using the Superscript III First-Strand Synthesis System (Invitrogen). For qPCR reactions, cDNA was diluted at a ratio of 1:5. Each qPCR reaction was performed in triplicate. Analysis of gene expression was performed using SYBR Green PCR Master Mix (Applied Biosystems) and Quant Studio 6 Real-Time PCR System. qPCR gene expression analysis was carried out using the comparative Ct method (ΔCT) using GAPDH as the reference housekeeping gene.

### Lymphatic endothelial cell isolation for flow cytometry and bulk RNA sequencing

For isolation of lung LECs, *Prox1-EGFP* reporter mice, in which all LECs are labeled with GFP, were injected with Alexa Fluor 647-conjugated isolectin (Invitrogen) for 12 min just prior to sacrifice. The lungs were then harvested and digested using collagenase and dispase in HBSS to generate a single-cell suspension for FACS, with additional staining using antibodies for CD31 (BioLegend 102418), lineage cocktail (BioLegend 133311), and EpCAM (BioLegend 118225). LECs were sorted using positive selection for GFP and CD31. Negative selection for EpCAM and a hematopoietic lineage marker cocktail was also performed. Total RNA from the LECs was isolated using a Qiagen RNeasy Mini Kit. For bulk RNA sequencing of LECs, at least 20 ng of total RNA from freshly harvested LECs was used for each replicate. For the 8-week CS time point, LECs were pooled from mice of the same experimental condition (n = 2–5) to have sufficient cell numbers and RNA quantity for analysis. Samples from the chronic CS exposure time points were not pooled. Libraries were prepared using the SMART-Seq v4 kit (Takara Bio), and bulk RNA sequencing was performed using Illumina NovaSeq 6000. Sample files were checked for sequence quality (FastQC v 0.11.9) and processed using the Digital Expression Explorer 2 (DEE2) (Ziemann et al., 2019) workflow. Adapter trimming was performed with Skewer (v0.2.2) (Jiang et al., 2014). Further quality control was carried out with Minion, part of the Kraken package (Davis et al., 2013). The resultant filtered reads were mapped to the mouse reference genome GRCm38/mm10 using the STAR aligner (Dobin et al., 2013) and gene-wise expression counts generated using the “-quantMode GeneCounts” parameter. After further filtering and quality control, the R package edgeR (Robinson et al., 2010) was used to calculate TMM normalization factors. FPKM and Log2 counts per million (CPM) matrices were quantified using these factors to normalize for library size. Principal component analysis and Pearson correlation values were quantified with the resulting Log2 CPM values. Gene enrichment analysis was run using g:profiler (Liao et al., 2019). Volcano plots were generated using the EnhancedVolcano R library (Blighe, K., *Rana*, S., and Lewis, M., 2018, https://github.com/kevinblighe/EnhancedVolcano).

### Human lung tissue

Slides of de-identified human lung tissue from patients with end-stage COPD or nonsmokers were collected and banked at Washington University School of Medicine under approved protocols, prior to use for immunohistochemical staining. COPD lung tissues (n = 10) were collected at the time of lung transplantation (WU IRB 201103213), and nonsmoker control tissues were collected from decedents that were not usable for lung transplantation at the Mid-America Transplant Organ Procurement Organization (n = 5) in St. Louis, Missouri. Lung tissues were formalin-fixed and paraffin-embedded in blocks. Fresh slides were cut prior to staining. Data mining of human single-cell RNA sequencing was performed by studying the publicly available data set generated by Sauler et al. (2022) (https://copdcellatlas.com) using the gene explorer and multi-gene query tools.

### Immunohistochemistry

Mice were sacrificed with CO_2_ exposure, and prior to harvest, the lungs were inflated with 4% PFA at a constant pressure of 25 cm H_2_O. The lungs were fixed in 4% PFA overnight at 4°. The lung tissue was then dehydrated by immersing it in a series of ethanol solutions of increasing concentrations and embedded in paraffin for sectioning. Six-micrometer sections were immunostained with antibodies for VEGFR3 (R&D Systems, AF743) and VCAM (Abcam, ab134047). Human lung tissue was stained with antibodies for podoplanin (D240, BioLegend, 75782-960) and VCAM (Abcam, ab134047) overnight at 4°. After washing, the slides were incubated with Alexa Fluor-conjugated secondary antibodies for 1 h at room temperature. Slides were treated with DAPI-containing Vectashield that was used on the tissue sections, and a cover slip was applied. Secondary antibodies alone were used on the negative control slides to check for autofluorescence of lung tissue. Immunofluorescence was performed either using a Nikon Eclipse microscope and NIS Elements software or a Zeiss SP8 confocal microscope and Leica software. Image analysis was performed using ImageJ.

### Statistics

Data are expressed as the mean ± SD. Statistical significance was determined by unpaired, 2-tailed Student’s t-test or ANOVA using GraphPad Prism software. *p* values of less than 0.05 were considered statistically significant.

### Study approval

All animal experiments were approved by the Institutional Animal Care and Use Committee (IACUC) of Weill Cornell Medicine and performed in accordance with relevant guidelines and regulations.

## Data Availability

The authors acknowledge that the data presented in this study must be deposited and made publicly available in an acceptable repository, prior to publication. Frontiers cannot accept a manuscript that does not adhere to our open data policies. The data presented in the study are deposited in the GEO repository, accession number GSE255250.
