# Supplementary material for: Lung lymphatic endothelial cells undergo inflammatory and prothrombotic changes in a model of chronic obstructive pulmonary disease
Source: Front Cell Dev Biol. 2024 Feb 19;12:1344070. doi: 10.3389/fcell.2024.1344070 (PMC10910060; doi:10.3389/fcell.2024.1344070)
Supplement: Supplementary file 1 [file DataSheet1.PDF]

Supplemental Figure 1

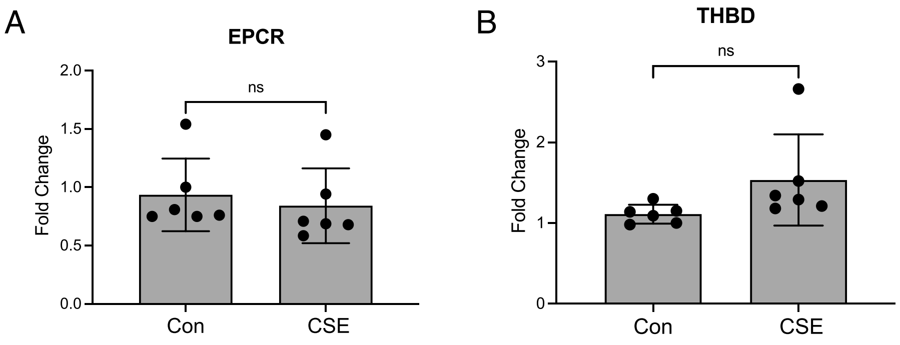

Supplemental Figure 2

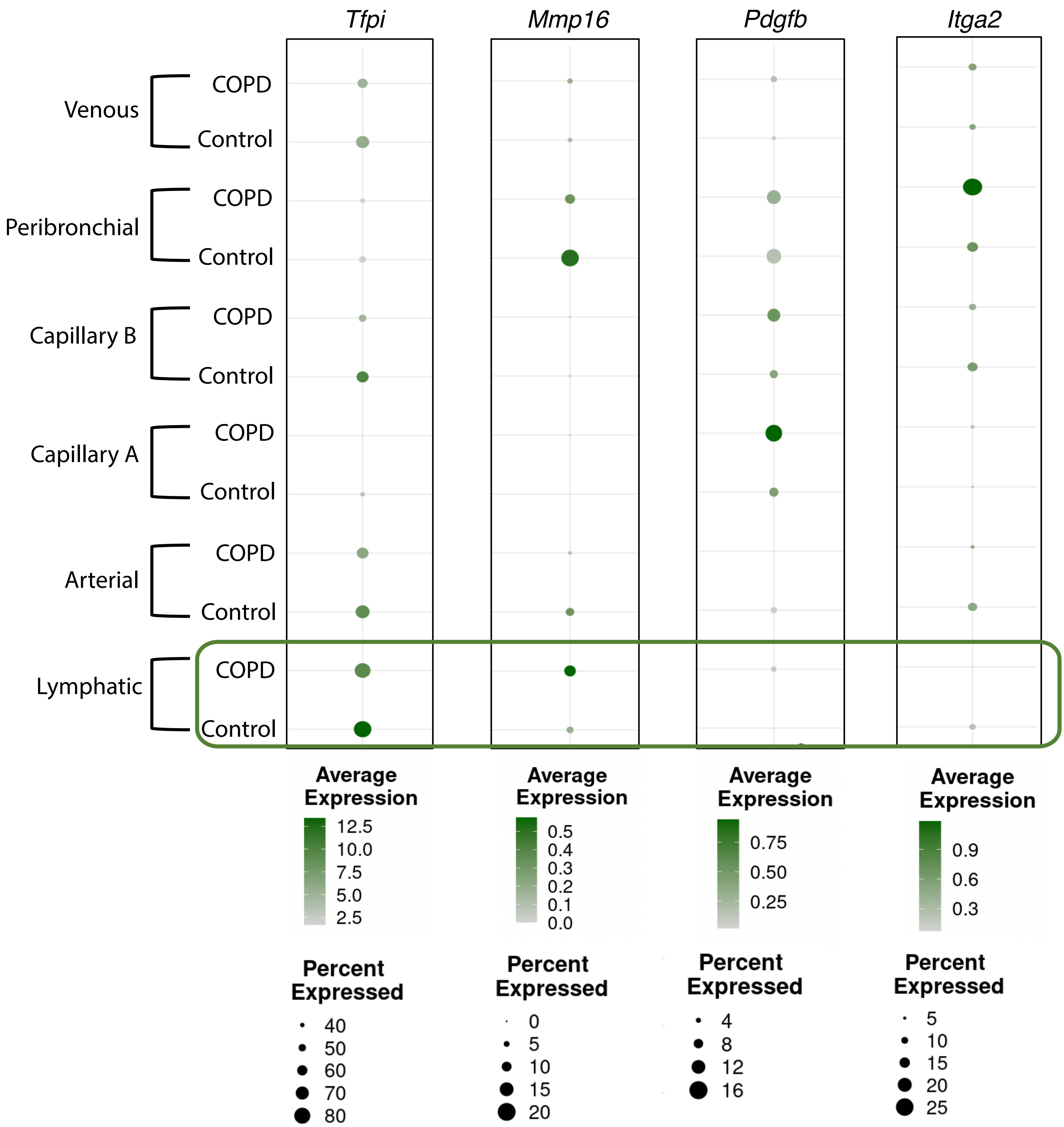

Supplemental Figure 1: Expression of EPCR (A) and THBD (B) by quantitative PCR using lysates from control or CSE-treated LECs and normalized to GAPDH. Values are expressed as means ± SD. P value calculated by Student's t test, ns = not significant. Data representative of 2 independent experiments performed in triplicate.

Supplemental Figure 2: Dot plot for gene expression from single cell RNA sequencing in endothelial cell subsets from COPD and control donor lungs. Dot size reflects the percentage of cells with gene expression, color corresponds to the magnitude of gene expression. Plots generated using online datamining tools from Sauler and McDonough et al, *Nature Communications* (2022) 13:494 (33) and http://copdatlas.com.
